# Supplementary material for: Upper limb muscle reflexes in real and virtual environments: Insights into sensorimotor adaptations
Source: PLoS One. 2025 Aug 13;20(8):e0330129. doi: 10.1371/journal.pone.0330129 (PMC12348997; doi:10.1371/journal.pone.0330129)

PLOS ONE

Upper Limb Muscle Reflexes in Real and Virtual Environments: Insights into Sensorimotor Adaptations

--Manuscript Draft--

**Manuscript Number:**	PONE-D-25-07247
**Article Type:**	Research Article
**Full Title:**	Upper Limb Muscle Reflexes in Real and Virtual Environments: Insights into Sensorimotor Adaptations
**Short Title:**	Reflex Adaptations in VR
**Corresponding Author:**	Ryan M Peters, Ph.D.<br>University of Calgary<br>Calgary, Alberta CANADA
**Keywords:**	Muscle Stretch Reflex; Hoffman Reflex; virtual reality; Electromyography; Electrodermal Activity; Motion Sickness
**Abstract:**	Introduction: The mechanisms influencing neuromuscular adaptations in the upper limb within dynamic environments remain understudied, especially when exposed to altered visual and emotional conditions such as those simulated in virtual reality (VR). Here we utilize VR to manipulate visual feedback, inducing motion sickness and modulating sympathetic arousal, while assessing adaptations in sensorimotor integration in the upper extremity using electrically evoked and muscle stretch reflexes.<br>Methods: Eighteen healthy young adults experienced four experimental conditions while sustaining submaximal activation of their flexor carpi radialis (FCR) muscle by isometrically supporting a weighted load: baseline real-world (Pre-VR), stationary VR (VR-BL), dynamic VR with motion perception via a virtual rollercoaster ride (VR-C), and post-VR following re-entry to the real environment (Post-VR). Muscle activity was monitored via electromyography (EMG), while reflex activity was assessed using electrical (H-reflexes) and mechanically induced (noisy tendon vibration; NTV) reflexes in the FCR. Additionally, electrodermal activity (EDA) and psychosocial indicators of motion sickness (subjective questionnaires) were measured throughout.<br>Results: H-reflex amplitude was suppressed during VR-C, which persisted into Post-VR; whereas NTV-reflexes were unaffected across conditions. Sympathetic arousal (e.g., EDA) and motion sickness symptoms increased significantly during VR-C compared to Pre-VR, but rapidly returned to baseline Post-VR. EMG within the target muscle (FCR) as well as in the brachioradialis was maintained across conditions, though increased activation was observed in the biceps brachii beginning at the onset of VR immersion (VR-BL).<br>Discussion: These findings suggest suppression of spinal excitability (H-reflex) when the perception of motion (VR-C) was added to a stationary VR experience. Meanwhile, muscle spindle sensitivity (NTV-reflex) remained consistent, highlighting potential fusimotor adaptations to maintain sensorimotor function under altered visual and emotional states. Persistent H-reflex suppression post-VR indicates lingering neuromuscular effects of immersive VR, underscoring the need for further exploration of VR's implications for rehabilitation and virtual training environments.
**Order of Authors:**	Daniel D Hodgson, M.Sc.
	Taha Butt, B.Sc.
	Brian H Dalton, Ph.D.
	Tyler Cluff, Ph.D.
	Ryan M Peters, Ph.D.
**Additional Information:**	
**Question**	**Response**
**Financial Disclosure**	Yes

Powered by Editorial Manager® and ProduXion Manager® from Aries Systems Corporation

Enter a financial disclosure statement that describes the sources of funding for the work included in this submission. Review the submission guidelines for detailed requirements. View published research articles from *PLOS ONE* for specific examples.<br><br>This statement is required for submission and **will appear in the published article** if the submission is accepted. Please make sure it is accurate.<br><br>**Funded studies**<br>Enter a statement with the following details:<br>• Initials of the authors who received each award<br>• Grant numbers awarded to each author<br>• The full name of each funder<br>• URL of each funder website<br>• Did the sponsors or funders play any role in the study design, data collection and analysis, decision to publish, or preparation of the manuscript?<br><br>Did you receive funding for this work?	
Please add funding details.<br>as follow-up to "**Financial Disclosure**<br><br>Enter a financial disclosure statement that describes the sources of funding for the work included in this submission. Review the submission guidelines for detailed requirements. View published research articles from *PLOS ONE* for specific examples.<br><br>This statement is required for submission and **will appear in the published article** if the submission is accepted. Please make sure it is accurate.	This research was funded by Natural Sciences and Engineering Research Council Discovery (RGPIN-2020-06068) and Idea-to-Innovation grants (I2IPJ 548811-2020), as well as a New Frontiers in Research Fund grant (NFRFR-2021-00199) awarded to RMP. DDH was additionally supported through a Natural Sciences and Engineering Research Council Doctoral Postgraduate Scholarship.

Powered by Editorial Manager® and ProduXion Manager® from Aries Systems Corporation

**Funded studies**<br>Enter a statement with the following details:<br>• Initials of the authors who received each award<br>• Grant numbers awarded to each author<br>• The full name of each funder<br>• URL of each funder website<br>• Did the sponsors or funders play any role in the study design, data collection and analysis, decision to publish, or preparation of the manuscript?<br><br>Did you receive funding for this work?"	
Please select the country of your main research funder (please select carefully as in some cases this is used in fee calculation).<br>as follow-up to "**Financial Disclosure**<br><br>Enter a financial disclosure statement that describes the sources of funding for the work included in this submission. Review the submission guidelines for detailed requirements. View published research articles from *PLOS ONE* for specific examples.<br><br>This statement is required for submission and **will appear in the published article** if the submission is accepted. Please make sure it is accurate.<br><br>**Funded studies**<br>Enter a statement with the following details:<br>• Initials of the authors who received each award<br>• Grant numbers awarded to each author<br>• The full name of each funder<br>• URL of each funder website<br>• Did the sponsors or funders play any role in the study design, data collection and analysis, decision to publish, or preparation of the manuscript?<br><br>Did you receive funding for this work?"	CANADA - CA
**Competing Interests**	The authors have declared that no competing interests exist.

Use the instructions below to enter a competing interest statement for this submission. On behalf of all authors, disclose any competing interests that could be perceived to bias this work—acknowledging all financial support and any other relevant financial or non-financial competing interests.<br><br>This statement is required for submission and **will appear in the published article** if the submission is accepted. Please make sure it is accurate and that any funding sources listed in your Funding Information later in the submission form are also declared in your Financial Disclosure statement.<br><br>View published research articles from *PLOS ONE* for specific examples.<br><br>**NO authors have competing interests**<br><br>Enter: *The authors have declared that no competing interests exist.*<br><br>**Authors with competing interests**<br><br>Enter competing interest details beginning with this statement:<br><br>*I have read the journal's policy and the authors of this manuscript have the following competing interests: [insert competing interests here]*<br><br>* typeset	
**Ethics Statement**<br><br>Enter an ethics statement for this submission. This statement is required if the study involved:<br><br>• Human participants<br>• Human specimens or tissue<br>• Vertebrate animals or cephalopods<br>• Vertebrate embryos or tissues	The University of Calgary's Conjoint Health Research Ethics Board approved all experimental protocols (REB17-2154)

• Field research<br><br>Write "N/A" if the submission does not require an ethics statement.<br><br>General guidance is provided below. Consult the submission guidelines for detailed instructions. **Make sure that all information entered here is included in the Methods section of the manuscript.**	

Powered by Editorial Manager® and ProduXion Manager® from Aries Systems Corporation

**Format for specific study types**<br><br>**Human Subject Research (involving human participants and/or tissue)**<br>• Give the name of the institutional review board or ethics committee that approved the study<br>• Include the approval number and/or a statement indicating approval of this research<br>• Indicate the form of consent obtained (written/oral) or the reason that consent was not obtained (e.g. the data were analyzed anonymously)<br><br>**Animal Research (involving vertebrate animals, embryos or tissues)**<br>• Provide the name of the Institutional Animal Care and Use Committee (IACUC) or other relevant ethics board that reviewed the study protocol, and indicate whether they approved this research or granted a formal waiver of ethical approval<br>• Include an approval number if one was obtained<br>• If the study involved *non-human primates*, add *additional details* about animal welfare and steps taken to ameliorate suffering<br>• If anesthesia, euthanasia, or any kind of animal sacrifice is part of the study, include briefly which substances and/or methods were applied<br><br>**Field Research**<br><br>Include the following details if this study involves the collection of plant, animal, or other materials from a natural setting:<br>• Field permit number<br>• Name of the institution or relevant body that granted permission	
**Data Availability**<br><br>Authors are required to make all data underlying the findings described fully available, without restriction, and from the time of publication. PLOS allows rare exceptions to address legal and ethical concerns. See the PLOS Data Policy and FAQ for detailed information.	Yes - all data are fully available without restriction

A Data Availability Statement describing where the data can be found is required at submission. Your answers to this question constitute the Data Availability Statement and **will be published in the article**, if accepted.<br><br>**Important:** Stating 'data available on request from the author' is not sufficient. If your data are only available upon request, select 'No' for the first question and explain your exceptional situation in the text box.<br><br>Do the authors confirm that all data underlying the findings described in their manuscript are fully available without restriction?	
**Describe where the data may be found in full sentences. If you are copying our sample text, replace any instances of XXX with the appropriate details.**<br><br>• If the data are **held or will be held in a public repository**, include URLs, accession numbers or DOIs. If this information will only be available after acceptance, indicate this by ticking the box below. For example: *All XXX files are available from the XXX database (accession number(s) XXX, XXX.*).<br>• If the data are all contained **within the manuscript and/or Supporting Information files**, enter the following: *All relevant data are within the manuscript and its Supporting Information files.*<br>• If neither of these applies but you are able to provide **details of access elsewhere**, with or without limitations, please do so. For example:<br><br>*Data cannot be shared publicly because of [XXX]. Data are available from the XXX Institutional Data Access / Ethics Committee (contact via XXX) for researchers who meet the criteria for access to confidential data.*<br><br>*The data underlying the results presented in the study are available from (include the name of the third party*	All data will be made available upon request to the corresponding author.

and contact information or URL).<br>• This text is appropriate if the data are owned by a third party and authors do not have permission to share the data.<br><br>* typeset	
Additional data availability information:	

Manuscript

Click here to access/download;Manuscript;Manuscript PLoS ONE_Hodgson et al .docx

Title:

Upper Limb Muscle Reflexes in Real and Virtual Environments: Insights into Sensorimotor Adaptations

Authors:

Daniel D Hodgson[1], Taha Butt[1], Brian H Dalton[2], Tyler Cluff[1,3], Ryan M Peters[1,3,4]

Author Affiliations:

[1]Faculty of Kinesiology, University of Calgary, Calgary, AB, Canada

[2]School of Health and Exercise Sciences, University of British Columbia Okanagan, Kelowna, BC, Canada

[3]Hotchkiss Brain Institute, University of Calgary, Calgary, AB, Canada

[4]Department of Biomedical Engineering, University of Calgary, Calgary, AB, Canada

Corresponding Author:

Ryan M. Peters, Ph.D.

Faculty of Kinesiology

University of Calgary

Calgary, AB, Canada

T2N 1N4

P: +1 (403) 220-7810

E: ryan.peters1@ucalgary.ca

Total Words:

9,553

Figures:

5

Tables:

1

ABSTRACT

Introduction: The mechanisms influencing neuromuscular adaptations in the upper limb within dynamic environments remain understudied, especially when exposed to altered visual and emotional conditions such as those simulated in virtual reality (VR). Here we utilize VR to manipulate visual feedback, inducing motion sickness and modulating sympathetic arousal, while assessing adaptations in sensorimotor integration in the upper extremity using electrically evoked and muscle stretch reflexes.

Methods: Eighteen healthy young adults experienced four experimental conditions while sustaining submaximal activation of their flexor carpi radialis (FCR) muscle by isometrically supporting a weighted load: baseline real-world (Pre-VR), stationary VR (VR-BL), dynamic VR with motion perception via a virtual rollercoaster ride (VR-C), and post-VR following re-entry to the real environment (Post-VR). Muscle activity was monitored via electromyography (EMG), while reflex activity was assessed using electrical (H-reflexes) and mechanically induced (noisy tendon vibration; NTV) reflexes in the FCR. Additionally, electrodermal activity (EDA) and psychosocial indicators of motion sickness (subjective questionnaires) were measured throughout.

Results: H-reflex amplitude was suppressed during VR-C, which persisted into Post-VR; whereas NTV-reflexes were unaffected across conditions. Sympathetic arousal (e.g., EDA) and motion sickness symptoms increased significantly during VR-C compared to Pre-VR, but rapidly returned to baseline Post-VR. EMG within the target muscle (FCR) as well as in the brachioradialis was

maintained across conditions, though increased activation was observed in the biceps brachii beginning at the onset of VR immersion (VR-BL).

Discussion: These findings suggest suppression of spinal excitability (H-reflex) when the perception of motion (VR-C) was added to a stationary VR experience. Meanwhile, muscle spindle sensitivity (NTV-reflex) remained consistent, highlighting potential fusimotor adaptations to maintain sensorimotor function under altered visual and emotional states. Persistent H-reflex suppression post-VR indicates lingering neuromuscular effects of immersive VR, underscoring the need for further exploration of VR's implications for rehabilitation and virtual training environments.

KEYWORDS: Muscle Stretch Reflex, Hoffman Reflex, Virtual Reality, Electromyography, Electrodermal Activity, Motion Sickness

INTRODUCTION

The ability to exhibit situation-appropriate motor control in dynamic environments is a testament to the intricate relationship between sensory inputs and motor outputs within the nervous system. This is orchestrated by a complex network of neural pathways, involving integration of sensory information (e.g., vision, proprioception, vestibular), under varying sympathetic and emotional states, to activate corresponding spinal and supraspinal neuromuscular outputs. While much research has focused on understanding these mechanisms within the lower limbs in the context of static balance, less attention has been given to the

neural adaptations that occur in the upper limbs during dynamic experiences involving altered visual, sympathetic, and emotional states.

Integral to motor control is sensory feedback from the periphery, such as from muscle and skin, which provides important information about the environment that can influence subsequent movement patterns. For example, myotatic stretch reflexes, mediated by muscle spindle receptors embedded within skeletal muscles, play a vital role in maintaining postural stability and coordinating muscle activity. Muscle spindles are fundamental stretch receptors that contribute to our sense of proprioception, with primary and secondary afferent nerves together capable of delivering precise information regarding the position (e.g., instantaneous length), velocity, and acceleration of the parent muscle [1–4]. Their unique efferent (fusimotor) innervation via gamma (γ-MN) and beta motor neurons (β-MN) enables direct control over their sensitivity from supraspinal and spinal circuits. Fusimotor drive enables the fine-tuning of spindle sensitivity and thereby directly governs motor responses to external perturbations [5–7]. Given the confluence of both peripheral and central inputs arriving in the spinal cord, context-dependent adaptations of the resulting stretch reflex may arise from altered descending commands that modulate the excitability of spinal alpha motor neurons (α-MN), interneurons, and Renshaw cells, and/or from changes in muscle spindle sensitivity via γ-MN activation [5,8–12].

In experimental settings, stretch reflexes can be elicited using mechanical stimulation such as tendon taps or vibrations, including noisy tendon vibration (NTV) [13–15]. The resulting muscle or tendon stretch from these techniques activates muscle spindles directly, providing insight into both their afferent sensitivity (influenced by γ-MN input) and the integrity of the

reflex arc including changes in pre- or post-synaptic excitability [16]. Alternatively, the reflex pathway may be activated using electrical stimulation of the peripheral nerve, an assessment known as the Hoffmann (H) reflex. The H-reflex bypasses muscle spindles and instead activates the afferent neurons along their axon, thereby offering a measure of spinal excitability and presynaptic inhibition [13,17,18]. So, measuring both NTV and H-reflexes allows for inferences about the sources of reflex adaptation. While numerous studies have incorporated either mechanical or electrical stimulation to assess neural activity, some researchers have compared reflex adaptation patterns between the two methods within the same experiment to infer whether condition-dependent adaptations occurred in muscle spindle afferent sensitivity (presumably via fusimotor activity), or within the spinal cord via altered pre- or post-synaptic excitability [e.g., 13,19,20].

In real-world environments, the excitability of the stretch reflex pathway appears to modulate alongside conditions such as postural configuration, the risk or consequence of a postural disturbance, and with sensory cues such as visual feedback. For instance, soleus H-reflex amplitude was reduced when standing compared to sitting, even when the background muscle activity was equivalent between conditions [21]. In fact, H-reflex attenuation corresponds with altered postural stability such as when changing position from lying to sitting to standing [18,22] or from quiet standing to standing on unstable terrain [23]. Furthermore, Sibley et al. [24] demonstrated that H-reflexes decrease in amplitude when subjects stand at the edge of a height platform, suggesting potential alterations in pre-synaptic inhibition. Other studies have shown similar findings of H-reflex suppression under conditions of heightened postural threat [25,26]. This may be an innate executive control existing to prevent excess

movements or overreactions to trivial perturbations that could lead to harm. Conversely, studies examining mechanically evoked reflexes have reported facilitated responses during similar real-world threatening conditions [19,27,28].

For example, Horslen and colleagues [19] observed the strengthening of tendon stretch reflexes when either the consequence (subjects stood at the edge of a 3.2 m platform) or the likelihood (the platform was occasionally tilted) of a fall was increased. This suggests a favoring of postural reflexes when the cost or risk of falling is high. More recently, they used a similar experimental design to demonstrate the significant enhancement of short and medium-latency stretch reflexes (via ramp-and-hold stretches) and tendon-tap reflexes during a height-induced postural threat [28]. Furthermore, the SLR–velocity relationship increased by 36.1% during the high threat condition, providing evidence of heightened dynamic muscle spindle sensitivity during postural threat. While these studies were limited to reflexes in the lower limb during standing, similar findings have been reported in the upper extremities. For example, Nafati et al. [20] reported an attenuation of H-reflexes and an excitation of mechanical reflexes in the extensor carpi radialis during the performance of a cognitively demanding task, suggesting that spindle sensitivity may similarly adapt in response to increased cortical demand or postural threat. Enhancing muscle spindle sensitivity during periods of stress or uncertainty may represent some distinctive protective mechanism to maintain or elevate the overall integrity of the stretch reflex, improving the chance of a successful correction if necessary – for example, in response to increased task demands or heightened likelihood or cost of an error, such as the risk of injury from a fall [19].

Virtual reality (VR) presents a unique tool to study motor control in immersive and controlled environments. VR equips researchers to manipulate visual feedback and induce sympathetic responses (e.g., changes in electrodermal activity; EDA) in a safe and practical manner while monitoring neural responses in real-time. Altered postural control and physiological/psychological indicators of stress seem to manifest similarly in real and VR settings [29–32]. Cleworth et al. [29] used real and VR environments matched in visual scale and detail to compare changes in standing postural control, EDA, and psychosocial states (e.g., subjective fear and anxiety) while participants stood at real and virtual heights. They observed similar adaptations across all measures, highlighting the utility of VR in replicating real-world threats and similarly modulating physiological parameters. Similarly, VR environments that alter the visual experience and/or sympathetic arousal appear to influence spinal reflexes as well [13,33].

Recently, Hodgson et al. [13] reported reduced H-reflex amplitude in virtual conditions of standing at both low and high heights compared to real environment standing at ground level, while NTV-induced reflexes remained unchanged. We proposed that fusimotor drive may elevate muscle spindle sensitivity, thereby contributing to the maintenance of stretch reflex amplitudes when spinal excitability (H-reflex) is reduced. We also noted that because there were no statistical differences in either reflex across VR height conditions, that the altered visual environment alone was sufficient to suppress H-reflexes. In a similar study, Grosprêtre and colleagues [33] used VR to simulate standing at ground level, atop a building, and falling from the building. H-reflex amplitudes were significantly dampened during their most stressful condition, falling from the building, although in contrast to Hodgson et al. [13], they observed no difference in H-reflexes between standing in the real environment compared to standing in

VR at either ground level or at height. Notably, this study did not monitor mechanically evoked reflexes. Despite this contradictory finding, complex visual states have been shown to depress H-reflex amplitudes [e.g., 32,34,35] and increase muscle spindle activity [36,37].

VR has been reported to induce symptoms of motion sickness, sometimes referred to as VR sickness or cybersickness. Notably, VR sickness, as well as deficits in proprioception [limb position sense; 38] and cognitive speed [39], have been reported to persist immediately following return to the real environment. It is unclear whether spinal reflexes exhibit such persistence following the return from VR – applicable research seems to focus on adaptations when entering VR while overlooking the reemergence to the real environment [13,33].

So, while progress has been made, the neuromuscular adaptations that occur, particularly in the upper extremities and in conditions altering visual feedback and sympathetic arousal (e.g., VR), are not thoroughly described. The primary objective of this study was to characterize the neural adaptations of upper limb reflexes induced by manipulating the visual environment and sympathetic arousal. We used electrical (H-reflex) and mechanical (NTV) stimulation to discern global (spinal excitability) and fusimotor (muscle spindle-dependent) changes in reflex behaviour under these different visual feedback conditions. To appropriately address this objective, we aimed to use VR to investigate whether visual feedback, without (VR-BL) or with (VR-C) the perception of motion, alters physiological (electrodermal activity) or psychosocial (motion sickness questionnaire) indicators of stress compared to two conditions in the real environment (Pre-VR and Post-VR). It was predicted that: (i) similar to in the lower limb during conditions of elevated sympathetic stress [13], H-reflex amplitudes in the upper limb would attenuate in VR and become the weakest in the most stressful (VR-C) condition, (ii)

mechanically evoked stretch reflex amplitudes would remain unaltered [13] under conditions of manipulated visual feedback and motion perception in the VR conditions, (iii) physiological indicators of stress and psychosocial indicators of motion sickness would be greatest in the VR-C condition, and (iv) VR-induced adaptations of physiological and psychosocial stress as well as the H-reflex would persist throughout the washout (Post-VR) condition upon returning to the real environment [38,39].

METHODS

Subjects

18 healthy young adults (mean age: 19.2 years; SD: 1.4 years; 9 females) were recruited for this study using a convenience sampling method. Participants entered the study with limited or no previous VR experience, were free from musculoskeletal injury in the upper limbs, and had no diagnosed neurological conditions which could impact experimental outcomes, such as multiple sclerosis or spinal cord injury. Written informed consent was obtained by all participants prior to taking part in this study. Recruitment for this study occurred from May to August 2024, and the University of Calgary’s Conjoint Health Research Ethics Board approved all experimental protocols.

Procedure

Experimental Setup

Following informed consent procedures, participants were equipped with electromyography (EMG), EDA, and electrical stimulation electrodes, as well as a custom-built

wearable tendon vibrator. Participants were then seated comfortably in a fully adjustable electrical reclining chair, manipulated such that their right arm rested on the armrest with a 90-degree angle at the elbow joint. The chair was adjusted forwards or backwards to achieve an appropriate distance from a hand crank lever, positioned next to the chair, which could be raised and held comfortably in their supinated right palm while maintaining a relaxed position (Figure 1A). Wrist angle and muscle activity in the FCR were monitored visually by the researchers throughout the experiment.

Figure 1: Experimental setup and raw data. **A:** Participants sat in an adjustable dental chair, donning a VR headset, EMG and EDA electrodes, nerve stimulation electrodes and a custom tendon vibrator. Background muscle activity was maintained by having them hold against a crank lever to counteract the torque applied from a set of pre-determined weighted plates. **B:** Example of the VR scene just prior to starting the rollercoaster, which was the scene for VR-BL condition. Asterisk at the crest of the first drop indicates the location where stimulation (H-reflexes or NTV) commenced. C: Exemplar 10s raw data traces during NTV stimulation, from top to bottom: Electrodermal Activity (EDA), Biceps Brachii EMG (BB), Brachioradialis EMG (BR), Flexor Carpi Radialis EMG (FCR), Tendon Vibrator Acceleration (Accel.) and motor command signal (Mtr. Cmd.).

Determination of Background Load Level

Consistent submaximal background FCR activation was encouraged to improve the signal-noise ratio of the H-reflex. Submaximal isometric contractions (10-30% of maximum activation) have been shown to increase the successful observation of FCR H-reflexes, from 35%

of participants at rest to 95% of participants during contraction [40]. It was crucial to obtain strong H-reflexes at the beginning of this study as their amplitude was predicted to decrease during the experiment. Improving the strength and success rate of observing an H-reflex enabled direct comparison of H-reflexes across conditions. This was achieved by placing an appropriate weighted load on the crank lever to be held by the participant. The load (eliciting 5–10% EMG%MAX in the FCR) was determined by first having participants perform three maximum voluntary contractions (MVC's), separated by 60 seconds, via manual muscle tests for the FCR: a researcher stabilized the participant's forearm in a fixed position against the armrest to isolate the wrist flexor muscles while applying a downward pressure on their supinated palm. Participants were instructed to exert maximal effort while flexing their wrist to counteract the downward pressure applied by the researcher. Each MVC effort was sustained for 3–5 seconds during which verbal encouragement was provided. EMG from the FCR muscle during the 3 MVC's was processed by removing the DC offset, rectifying, and taking the root mean squared (RMS) average (50 ms windows). The maximum EMG value was identified and represented 100% FCR muscle activation. Next, weighted plates (0.25–1 lb increments) were added to the crank lever until FCR EMG%MAX remained constant between 5–10% during a sustained isometric lever hold. This crank lever load was then used for the remainder of the experiment. Wrist angle was monitored for any deviations from this fixed position and verbal feedback was provided if adjustments were required.

Determination of Electrical Stimulation Intensity

A one-time electrical stimulation recruitment curve procedure preceded the experimental procedure to determine the maximum peak-to-peak (P2P) amplitude and

corresponding stimulus intensity values for the H-reflex (H_{max}) and M-wave (M_{max}) in the FCR muscle. Since these electrically evoked values vary between individuals, establishing an appropriate stimulation current for the testing protocol specific to each participant is necessary. To accomplish this, participants were comfortably positioned in the adjusted chair with their arm relaxed at a 90-degree elbow angle. Their supinated right hand extended from the armrest and held the loaded crank lever (eliciting 5–10% FCR EMG%MAX) in a horizontal position while 1 ms square-wave pulses of electrical stimulation were delivered over the median nerve proximal to the elbow and medial to the biceps brachii muscle. Stimulations were delivered at least 10 seconds apart to mitigate risk of interference from presynaptic inhibition [18,41]. To limit fatigue, a researcher supported the crank lever, allowing the participant to relax, between electrical deliveries. The H_{max} and M_{max} were defined as the greatest P2P amplitude produced by the smallest amount of current. Stimulation current began at 5 mA with subsequent 1–2 mA adjustments until an H-wave was detected for the FCR. Current increases continued until the H-wave amplitude began to descend with increasing current, at which point the current was dialed back by increments of 0.5–1 mA until the H_{max} was determined. To locate the M_{max}, 2–5 mA current increments resumed until a plateau was observed in the rising M-wave. To ensure that maximal M-waves were consistently generated, 20% was added to the stimulation current required for producing M_{max}. This supramaximal stimulation current for the M_{max} and the current required to elicit H-max were used for the electrical stimulation reflex assessments throughout the study. These recruitment curve procedures were completed within approximately 5 minutes, with 15–30 total electrical pulses delivered, and were followed by two minutes of rest prior to commencing the testing interventions.

Testing Interventions

During the subsequent testing protocol, participants experienced four test conditions while maintaining a comfortable seated posture throughout. Each of the four conditions comprised four 60-second periods of stimulation: two periods of mechanical stimulation and two periods of electrical stimulation performed in randomized alternating order. Mechanical stimulation consisted of NTV (0–100 Hz) over the FCR tendon near the wrist (evoking NTV-reflex); electrical stimulation was delivered to the median nerve using stimulation current intensities evoking H-reflexes and M-waves (see above). With approximately 10-seconds between deliveries, a total of seven electrical reflexes were elicited during each 60-second electrical stimulation period in randomized order: five targeting H_{max} and two targeting M_{max}. The crank lever with a predetermined load (eliciting 5–10% of maximum FCR activation; see above) was held in a static horizontal position during all stimulation periods with 60-second breaks allotted between each epoch. Verbal feedback was provided by the researchers to correct for any deviations in crank lever position, which was monitored visually. Each condition (including all four stimulation periods) was proceeded by the delivery of a Motion Sickness Assessment Questionnaire (MSAQ) [42] and the Borg CR-10 scale for measuring subjective rating of perceived exertion (RPE) [43].

Experimental Procedures and Conditions

The four conditions commenced with the first performed in the real environment ("Pre-VR"), followed by two conditions (one before and one during a virtual rollercoaster ride) in the VR environment (VR-baseline: "VR-BL", and VR-coaster: "VR-C"), and concluded with a final real-

world condition ("Post-VR"). A two-minute break separated each condition enabling the participant to release the crank lever and relax. Following Pre-VR, participants were introduced to the virtual environment. They donned an HTC Vive Pro 2 headset (HTC Corporation, Taiwan) with the fitment and view adjusted based on participant feedback until the clearest picture was achieved. A VR game "Epic Roller Coasters" was used for the VR environment, with the "Tropical Island" rollercoaster track selected for all participants. This track provides a rollercoaster ride approximately 60-seconds long, matching the timeframe for the stimulation periods in this study. Thus, participants completed four laps (e.g., 2× for H-reflexes and 2× for NTV reflexes) of the rollercoaster track during the VR-C condition. Upon transition into VR, participants were seated in a stationary rollercoaster cart at the beginning of the track, where an immersion protocol familiarized them with the virtual environment. They were asked to: 1) describe your surroundings, 2) locate the ocean to your right, 3) locate the large boulder to your left, 4) locate the treetops above you, and 5) locate the rollercoaster tracks beneath you. A monitor displaying the participant's field of view was positioned in the lab for reference. Following the familiarization protocol and a 60-second break, the two VR conditions commenced in sequence. VR-BL was performed first with the participant stationary at the beginning of the rollercoaster track (Figure 1B). The rollercoaster was then set in motion for the VR-C condition and the participant perceived their coaster cart climb an initial summit, taking approximately 60-seconds. The first, and each subsequent, stimulation period for the VR-C condition was initiated as the cart rounded the summit and began a steep rapid descent (see asterisk in Figure 1B). The remaining rollercoaster ride persisted approximately 60-seconds, incorporating fast-paced sharp turns, rises, drops, and loops, taking the rider around an interactive tropical island scene

immersed with trees, birds, and ocean waves. Four laps were completed in sequence, with interleaved stimulation periods (NTV or electrical) each encapsulating one lap. Following the fourth and final lap, the participant returned to the starting position at the beginning of the rollercoaster track where the MSAQ was delivered, completing the VR-C condition. The VR headset was removed, reintroducing the participant back to the real-world environment to complete the final condition, Post-VR. Each of the four conditions lasted approximately eight minutes including four 60-second stimulation periods (two each of NTV and electrical), each separated by a 60-second rest period, and the MSAQ (~60-seconds).

Data Collection

Electrical Nerve Stimulation

Electrical stimulation of the median nerve was applied to generate H-reflexes and M-waves in the FCR muscle. H-reflexes are initiated along the peripheral afferent nerve and therefore differ from the spindle-generated NTV-reflexes. Following previous studies, H-reflexes were used alongside mechanically evoked tendon reflexes to postulate site-specific neural adaptations [13,19,27] – H-reflexes assess the reflex circuitry from the point of stimulation at the peripheral nerve onward, while NTV-reflexes encompass the entire circuit, beginning with muscle spindle excitation. The M_{max} was used to monitor for changes in motoneuron activation (e.g., movement of a stimulating electrode) and peripheral excitability (e.g., sarcolemma excitability, neuromuscular propagation, or altered electrode position in relation to underlying muscle fibers) between conditions, which could confound H-reflex observations. Surface electrodes (MediTrace, Canada) were used for the anode and cathode. They were placed in

sequence along the path of the median nerve, with the cathode approximately 5–7 cm proximal to the elbow joint just medial to the BB muscle belly and the anode centred 2 cm distally. A 3D printed rectangular block, custom designed to house the electrode connectors, was secured over the stimulation electrodes using a tensor bandage. By gently compressing the electrodes towards the median nerve, this method enabled optimal access and more consistent stimulation. A constant-current stimulator (STMISOLA, Biopac, USA) was used to deliver square-waveform electrical pulses (1 ms duration) using custom LabVIEW software (National Instruments, USA). Stimulator current (1–100 mA) was manually adjusted for each stimulus to the predetermined output for producing the desired physiological response.

Noisy Tendon Vibration

Muscle spindle-generated reflexes in the FCR muscle were evoked via mechanical stimulation of its tendon at the wrist. A custom-made wearable vibrator, secured to the distal forearm using an elastic strap (2 cm proximal to the wrist crease with the vibrating motor positioned along a direct line connecting the medial epicondyle and the scaphoid bone), was used to deliver NTV (0–100 Hz) to the FCR tendon. The elastic strap was secured in position and held at a snug but comfortable tension prior to performing any study procedures and remained in place for the entire experiment. The device consists of a custom 3D printed housing with onboard motor (Tactile Labs, Canada), amplifier, and accelerometer (Analog Devices Ltd., USA). Motor command signals were generated using custom LabVIEW software (National Instruments, USA) and output at 10 kHz from a real-time PXI system (PXIe-1062Q chassis; PXI-8105 embedded controller) with a multifunction data acquisition card (PXI-6289) and A/D board (BNC-2090).

Electromyography

Muscle activity was monitored throughout the study using surface EMG. Prior to surface electrode placement, the skin overlaying the muscles of interest was prepared as appropriate (e.g., shaved and cleaned with alcohol swabs). Two surface electrodes (Ambu, Malaysia) were applied to the skin, 2 cm centre-to-centre spacing, over the right FCR, brachioradialis (BR), and biceps brachii (BB). Electrodes were placed parallel to muscle fiber orientation over the thickest region of the muscle belly. For FCR, this was located approximately 1/3 of the distance along a direct line between the medial epicondyle and the radial styloid process. The BR electrodes were placed approximately 3 cm lateral to the FCR electrodes along the radial aspect of the antebrachium. The BB electrode position was approximately 1/3 of the distance from the cubital fossa to the acromion process in the midline of the muscle belly [44]. To ensure appropriate positioning of surface electrodes, muscle bellies were visualized and palpated during isometric contraction. A reference electrode was placed over the olecranon process. EMG was analog high-pass filtered at 30 Hz, amplified with a gain of 500 (NeuroLog NL844 pre-amplifier and NL820A isolated amplifier; Digitimer Ltd., England), and sampled at 5 kHz using a Power 1401 A/D board and Spike2 v10.13 (Cambridge Electronic Design, England). A 4th order low-pass IIR digital filter at 1000 Hz was applied offline to remove any high-frequency noise (MATLAB, Mathworks, USA).

Electrodermal Activity

The galvanic skin conductance (e.g., sudomotor response) was monitored throughout the study by measuring electrodermal activity (EDA), providing an objective measurement of

sympathetic arousal [45]. For each condition, EDA during NTV and electrical stimulation was assessed separately rather than pooled together to account for any influence of stimulation method on arousal. Two surface electrodes (Ambu, Malaysia) were placed on the thenar eminence of the left hand [13]. A Skin Conduction Unit (Cambridge Electronic Design, England) recorded EDA with a sampling rate of 5 KHz using a Power 1401 A/D board and Spike2 v10.13 (Cambridge Electronic Design, England)

Psychosocial Assessments and Questionnaires

A Motion Sickness Assessment Questionnaire (MSAQ) was delivered following each condition [42]. The MSAQ consists of 16 statements pertaining to the subjective feelings of gastrointestinal (e.g., "I felt sick to my stomach"), central (e.g., "I felt faint-like"), peripheral (e.g., I felt sweaty"), and sopite-related (e.g., "I felt annoyed/irritated") distress experienced during the condition. Each statement was read aloud by a researcher to which the participant verbally expressed their agreement on a 1–9 scale (1 = "Not at all"; 9 = "Severely"). This questionnaire has been demonstrated to have high reliability under conditions inducive of motion sickness [42]. Scores from each of the four identified dimensions of motion sickness (gastrointestinal, central, peripheral, and sopite-related) were analyzed separately as well as concatenated to achieve an overall measurement of motion sickness. Additionally, the Borg CR-10 RPE scale [43] was employed to assess participants' perceived exertion during the study. This simple and widely recognized tool is commonly used to gauge exertion levels in exercise testing, training, and rehabilitation and has been validated against objective measures of exercise intensity [46].

Data Analysis

MATLAB (MathWorks, USA) and SPSS software (version 26.0; IBM SPSS Statistics Inc., USA) were used for all analysis with α set to 0.05. General linear mixed effects models were used to determine the main effect of visual condition on dependent variables. This statistical model assessed the relationships between all conditions in real (Pre-VR and Post-VR) and virtual (VR-BL and VR-C) environments with pairwise estimates. When a significant main effect was found, we conducted pairwise contrasts using the Least Significant Difference (LSD) method. LSD balances the trade-off between Type I (false positives) and Type II (false negatives) errors, being more powerful and less conservative than other correction methods, which can help avoid Type II errors, especially in studies with smaller sample sizes or where effects are subtle [47]. However, we acknowledge that LSD does not strictly control for multiple comparisons as well as other corrections. Dependent variables in both real and VR environments include mean EDA (assessed independently during NTV and electrical stimulation), mean MSAQ responses (collapsed into each of the four identified motion sickness dimensions as well as pooled altogether for a general indicator of motion sickness), rectified root mean squared (RMS) background EMG in each muscle (FCR, BR, and BB), H-reflex P2P amplitude in the FCR, and NTV-reflex cumulant density P2P amplitude in the FCR. Cumulant density estimates are time-domain representations of the cross-spectral density computed between the input (vibration acceleration magnitude) and output (rectified EMG) signal, as described in detail previously [13,14]. For the input signal to all subsequent NTV analyses, we computed acceleration magnitude as the square root of the sum of squared x, y, and z axis accelerations (in units of G) [13]. Using vibration magnitude is favorable in this case, as the wearable device vibrates in

three-dimensions and we sought to fully capture this motion – this is unlike previous studies that used bulkier motors which moved only in one axis [e.g., 14]. Mean EDA during each type of stimulus delivery (electrical vs. NTV) was compared for each condition using paired t-tests to determine if participant arousal was consistent during H-reflex and NTV-reflex assessment. The mean time to peak for H-reflexes and NTV-reflex cumulant density will be measured as the latency between stimulus delivery and the maximum value of the initial peak of the bi-phasic reflex wave. Cohen's d effect sizes were calculated; magnitude of the effects may be interpreted as small (ES = 0.2), medium (ES = 0.5), or large (ES = 0.8) [48].

RESULTS

General results

Two potential participants were screened from the study as we were unable to ascertain a consistent H-reflex in their FCR. This aligns with previous reports of a 72-100% success rate for evoking a response in this muscle [18]. Of the remaining 18 participants, all displayed strong FCR H-reflexes via median nerve stimulation. Despite receiving primary innervation from different nerves, occasional muscle responses to H-reflex were observed in the BR and BB muscles, although these were generally weak and inconsistent compared to those in FCR. Thus, we focused our H-reflex analysis on the FCR as planned. EDA data from one participant was excluded due to equipment malfunction during the experiment. Additionally, data points exceeding +/- 2SDs from the mean were considered outliers and removed. This resulted in the exclusion of a participant's data from four additional averages in the final analysis of EDA, Hmax, and BB EMG, one from each of the four experimental conditions. Each of these cases were

irregularly high values. All remaining data points were within normal anticipated ranges consistent with previous research in our laboratory and were included in the analysis [13].

Electrically evoked reflexes

H-reflexes: There was a significant main effect for condition on H-reflex P2P amplitude (F(3, 6) = 3.99; p = 0.011) (Figure 4). Significant pairwise contrasts indicated a sustained reduction in H-reflex strength beginning during the VR-C condition, which evoked reflexes smaller than both Pre-VR (22.3%; t(64) = 2.428; p = 0.018; CI: 20.53 – 211.35; *d* = 0.41) and VR-BL (20.5%; t(64) = 2.202; p = 0.031; CI: 9.78 – 200.6; *d* = 0.45) conditions. This reduction persisted upon return to the real environment, with Post-VR also registering smaller H-reflexes than both Pre-VR (24.6%; t(64) = 2.668; p = 0.010; CI: 32.0 – 222.84; *d* = 0.37) and VR-BL (22.8%; t(64) = 2.443; p = 0.017; CI: 21.27 – 212.09; *d* = 0.41). There were no significant differences between real and VR baseline conditions (Pre-VR vs. VR-BL) or between VR-C and Post-VR. The mean time to peak for the H-reflex pooled across conditions was 18.8 ms.

M_{max}: Two supramaximal stimuli representing M_{max} were averaged within each condition. There were no significant differences in M_{max} P2P amplitude across any conditions. The mean time to peak after stimulus for the M_{max} pooled across conditions was 6.0 ms.

M-wave accompanying the H-reflex: H-reflexes were typically accompanied by a small submaximal preceding M-wave which was monitored across conditions. The main effect for condition on the accompanying M-wave was not statistically significant (F(3, 63) = 2.143; p = 0.104).

Figure 2: H-reflex results across visual conditions. **A:** Example of an H-reflex response from a single stimulation pulse, showing the artifact at time zero, and the H-reflex occurring within latencies of ~20-30 ms. **B:** Mean H-reflex peak-to-peak amplitudes for each subject across visual conditions. Boxplots show the interquartile range (IQR), red lines indicate mean values, and error bars represent the full range of observed values for each condition. Asterisks denote significance at the p = 0.01 (*), 0.01 (**), and 0.001 (***) levels.

Noisy tendon vibration reflexes

There was no significant main effect for condition on the P2P amplitude of the NTV-reflexes ($F(3, 68) = 0.78$; $p = 0.509$) (Figure 5). The mean time to peak after stimulus of NTV-reflexes was 24.6 ms and no differences were observed between any experimental conditions.

Figure 3: Noisy Tendon Vibration (NTV-reflex) results across visual conditions. **A:** Example of an NTV response from 120 s of vibration, showing a biphasic, short-latency response occurring within latencies of ~20-50 ms. **B:** Mean NTV peak-to-peak cumulant density for each subject across visual conditions. All other notations are identical to Figure 2.

Background muscle activity

The FCR was loaded with a weight targeting 5-10% of its EMG%MVC to optimize the signal-noise ratio of the reflex responses and to avoid the confounding factor of changes in muscle activity across conditions. Mean background EMG%MVC in the FCR was 8.1% and there were no differences between conditions ($F(3, 68) = 0.732$; $p = 0.536$)(Figure 3). Thus, the target muscle remained consistently active throughout the experiment. Similarly, there were no differences in EMG across any conditions for the BR conditions ($F(3, 64) = 1.092$; $p = 0.359$).

However, there was a main effect for condition on BB EMG (F(3, 64) = 4.018; p = 0.011). Pairwise contrasts demonstrated significantly greater BB EMG in all conditions compared to Pre-VR, including VR-BL (42.3%; t(64) = 2.712; p = 0.009; CI: 4.11 – 27.11; *d* = 0.67), VR-C (47.3%; t(64) = 3.036; p = 0.003; CI: 5.98 – 28.97; *d* = 0.75), and Post-VR (42.2%; t(64) = 2.705; p = 0.009; CI: 4.07 – 27.07; *d* = 0.67).

Figure 4: Perceived fatigue level and background muscle activity across visual conditions. **A:** Borg scale CR10 scores for each condition. **B-D:** RMS background muscle activity across each condition for FCR (‘target muscle’), BR, and BB, respectively. All other notations are identical to Figure 2.

Electrodermal activity

Mean EDA for each condition was ascertained during the assessment of NTV and H-reflexes independently to account for any influence of stimulation method on arousal. While mean EDA throughout each stimulation technique exhibited a similar trend across conditions, EDA was higher during electrical (H-reflex; M=14.5 μS, SD=7.5 μS) compared to mechanical (NTV; M=13.4 μS, SD=7.43 μS) stimulation (8.2%; t(16) = 2.5, p = 0.025, *d* = 0.635) when pooled across all conditions. The electrical stimulation itself may have transiently elevated EDA in some participants creating this discrepancy. To minimize potential confounding effects caused by the H-reflex test stimulus, we focus on reporting EDA measured during NTV delivery which was more consistent throughout trials and across participants. As demonstrated in (Figure 2), EDA was elevated in all conditions compared to the real environment baseline condition (Pre-VR), reaching significance when the virtual rollercoaster was set in motion (VR-C; 42.6%; t(60) =

2.543; p = 0.014; CI: 1.000 – 8.378; *d* = 0.63) and falling just short of significance after returning to the real environment (Post-VR; 30.2%; t(60) = 1.780; p = 0.080; CI: -0.411 – 7.058; *d* = 0.45).

Figure 5: Perceived motion sickness and sympathetic activity across visual conditions. **A:** Mean MSAQ questionnaire scores for each subsection (grey scale) and combined (red). **B:** Mean EDA level across each of the visual conditions. All other notations are identical to Figure 2.

Psychosocial Assessments and Questionnaires

The MSAQ questionnaire was administered at the conclusion of all four conditions. There was a significant main effect for condition (F(3, 68) = 11.9; p < 0.001) for overall motion sickness (all 16 items combined). When divided into separate questionnaire components, significant main condition effects were discovered for each of gastrointestinal, central, peripheral and sopite-related indicators of motion sickness. For the pooled result, as well as for each separate component, pairwise contrasts indicated significantly elevated motion sickness when both visual and motion perception were modulated during VR-C compared to all other conditions. Notably, there was no difference in overall, nor in any particular indicator of motion sickness, between the two real (Pre-VR and Post-VR) conditions, indicating that these symptoms did not persist upon re-emergence from VR. MSAQ responses are depicted in Figure 2 and statistical differences are summarized in Table 1.

Finally, there was a significant main effect on condition for subjective muscle exertion reported using the Borg-scale (F(3, 68) = 3.368; p = 0.023). Pairwise contrasts revealed that significant elevations in perceived muscle exertion occurred during both VR-C (t(68) = 2.864; p =

0.006; CI: 0.286-1.602; d = 0.70) and Post-VR (t(68) = 2.527; p = 0.014; CI: 0.175-1.491; d = 0.62) compared to Pre-VR.

Table 1: Summary of significant pairwise comparisons for perceived motion sickness (Motion Sickness Assessment Questionnaire; MSAQ) and muscle exertion (Borg CR-10 scale). MSAQ data are pooled (Overall) and separated into four indicators of motion sickness: gastrointestinal (G), central (C), peripheral (P), and sopite-related (S).

Measure	*Main Effect*	*Condition Comparison*	*% Change*	*t(68)*	*p-value*	*Confidence Interval (CI)*	*Effect Size (Cohen's* d*)*
MSAQ – Overall	(F(3, 68) = 11.9; p < 0.001	VR-C – Pre-VR	132.1%	5.132	< 0.001	10.68 – 24.27	1.24
		VR-C – VR-BL	126.1%	5.03	< 0.001	10.33 – 23.93	1.21
		VR-C – Post-VR	91.3%	4.305	< 0.001	7.87 – 21.46	1.10
Gastrointestinal Indicators (G)	(F(3, 68) = 11.096; p < 0.001)	VR-C – Pre-VR	188.3%	4.911	< 0.001	13.28 – 31.47	1.20
		VR-C – VR-BL	192.1%	4.945	< 0.001	13.44 – 31.62	1.21
		VR-C – Post-VR	117.7%	4.064	< 0.001	9.45 – 27.61	1.05
Central Indicators (C)	(F(3, 68) = 11.304; p < 0.001)	VR-C – Pre-VR	147.9%	5.029	< 0.001	10.58 – 24.49	1.47
		VR-C – VR-BL	131.1%	4.781	< 0.001	9.71 – 23.62	1.39
		VR-C – Post-VR	107%	4.356	< 0.001	8.23 – 22.14	1.34
Peripheral Indicators (P)	(F(3, 68) = 5.5; p = 0.002)	VR-C – Pre-VR	70.6%	3.167	= 0.002	4.57 – 20.13	0.83
		VR-C – VR-BL	96%	3.748	< 0.001	6.83 – 22.39	1.00
		VR-C – Post-VR	54.3%	2.692	< 0.001	2.72 – 18.27	0.75
Sopite-related Indicators (S)	(F(3, 68) = 7.028; p < 0.001)	VR-C – Pre-VR	89.4%	4.255	< 0.001	6.23 – 17.23	1.18
		VR-C – VR-BL	62.6%	3.472	= 0.001	4.07 – 15.07	0.94
		VR-C – Post-VR	54.8%	3.192	= 0.002	3.30 – 14.30	0.95
BORG CR-10	(F(3, 68) = 3.368; p = 0.023)	Pre-VR – VR-C		2.864	= 0.006	0.29 – 1.60	0.70
		Pre-VR – Post-VR		2.527	= 0.014	0.18 – 1.49	0.62

DISCUSSION

Summary

The primary purpose of this study was to explore the adaptations of upper limb muscle reflexes under conditions of varying sensory feedback in the real and virtual realms. We observed a substantial weakening of the H-reflex when a motion-induced threat was perceived

in the form of a virtual rollercoaster ride. This adaptation did not initially emerge when simply transitioning into a stationary VR environment but materialized in the more dynamic VR-C condition and was sustained following re-emergence into the real environment. Meanwhile, NTV-reflex strength remained steady throughout all manipulations. Background EMG in the FCR and BR was unchanged across conditions, although there was an increase in BB activity in all conditions following Pre-VR. To appropriately address our primary research objective, we also aimed to use VR to investigate whether altered visual feedback, with or without the perception of motion, impacts physiological or psychosocial indicators of stress. Following a similar timeline to the observed H-reflex adaptations, a small physiological arousal response was identified upon entry into VR which escalated to statistical significance only after motion perception was introduced during the VR-C condition. Similarly, subjective reports of motion sickness were significantly greater during VR-C compared to all other conditions, both with responses concatenated as well as when separated into four distinct sub-categories. Finally, perceived muscle exertion was elevated during the VR-C and Post-VR conditions.

H-reflex and NTV-reflex modulation

The primary objective of this research was to examine the neuromuscular adaptations to the altered sensory feedback presented with both stationary and motion-simulating VR experiences. Interestingly, H-reflex strength was not impacted by transitioning into a stationary VR scene (VR-BL) but was significantly decreased when the virtual rollercoaster was set in motion (VR-C), an adaptation that persisted beyond the transition back into the real environment (Post-VR). Meanwhile, NTV-reflexes remained constant during transition into VR, throughout the VR experience, and after re-emergence into the real environment.

H-reflexes: In contrast to our prediction, the H-reflex was consistent between Pre-VR and VR-BL conditions. This is contrary to our previous finding, which highlighted that transitioning into a stationary VR environment while standing was sufficient to supress H-reflex strength in the soleus muscle [13]. It was postulated that, given the otherwise similar nature of the two conditions, the neural control of standing is altered in VR. Considering the present findings though, it seems that this adaptation may be task or posture dependent – despite maintaining a constant level of activation, the loaded upper limb muscles here did not contribute to maintaining stability or balance in a seated position as the lower extremity muscles did during standing in the previous study. So, the neural adaptations that occur alongside the visual perturbation of VR immersion may be task or muscle dependent, or at least primarily targeted toward the body regions most involved in the task. However, one other study has reported on H-reflex adaptations using VR and they observed no differences between real and virtual conditions, despite measuring from the soleus muscle during stance [33]. Nonetheless, complex visual states have been shown to depress H-reflex amplitudes [e.g., 32,34,35]. Further research is needed to conclusively determine how VR affects spinal excitability dependent on posture, task, and body region.

Consistent with our prediction, H-reflex amplitude was significantly reduced during the VR-C condition compared to Pre-VR. This finding aligns with previous studies which have demonstrated H-reflex suppression during their most arousing conditions in real [20,25,26] or virtual [13,29,30,32,33] environments. This general agreement indicates that increased sensory input can downregulate reflexive motor responses, likely due to heightened CNS activity leading to an increase in descending spinal inhibitory commands. In the current study, this adaptation

coincided with the introduction of motion perception to the stationary VR scene and concurrent elevations in subjective motion sickness and objective sympathetic stress. The reduction in H-reflex amplitude during VR-C may reflect a protective mechanism in response to the perceived threat, where descending spinal excitability is downregulated to prevent excess movements or overreactions to trivial perturbations that could lead to harm.

The reduction in H-reflex strength alongside the VR-C condition persisted even after returning to the real environment (Post-VR). To our knowledge, this is a novel finding – previous research investigating neuromuscular responses in VR seems to focus solely on adaptations occurring prior to and during VR exposure while overlooking the aftereffects. The persistent reduction during Post-VR suggests a residual effect of the VR experience on the CNS. This could be due to fatigue in the neural circuitry, but considering that EDA only marginally recovered during Post-VR, it likely is due to elevated sympathetic arousal that is either lingering from the VR experience or that has been re-established due to the visual perturbation associated with the return from VR.

<u>NTV-reflexes:</u> Aligning with our prediction, NTV reflexes remained constant during the transition from the real environment to both stationary and dynamic VR conditions, as well as post-VR. This agrees with Hodgson and colleagues [13], who reported consistent NTV-reflexes in the soleus during standing throughout real (eyes open and eyes closed) and virtual (low and high platform height) conditions of varying visual feedback and perceived threat. Given that this result coincided with weakened H-reflexes during their VR conditions, they posited that muscle spindle sensitivity becomes enhanced when spinal excitability (H-reflex) is reduced, thereby facilitating the maintenance of the overall stretch reflex integrity. A similar trend has emerged in

the current study, particularly during the VR-C and Post-VR conditions when H-reflexes were weakened while NTV-reflexes remained unaffected.

Background muscle activity and neuromuscular fatigue

To standardize background EMG in the FCR, participants supported a crank lever loaded to elicit 5–10% of their maximal FCR activity. We observed a mean of 8.1% maximal FCR output with no significant differences between conditions, indicating stable muscle activity throughout the experiment Consequently, no differences in EMG were noted in the FCR or BR, indicating that the observed changes in the H-reflex were not heavily influenced by the level of muscle activity in these muscles. However, the BB showed a significant increase in EMG immediately after the baseline (Pre-VR) condition, which remained elevated throughout (VR-BL, VR-C) and following (Post-VR) the VR experience. This increased BB activity may be attributed to increased tensing or co-contraction influenced by the sensory perturbation of VR. However, co-contraction, which requires the competing actions of an agonist-antagonist pair, was not directly measured in this study. Still, increased stiffness has been observed when postural control is either compromised [49] or threatened [50,51], and with visual manipulations such as emotion-provoking images [52,53] and VR [13]. However, most of these studies report increased co-contraction of muscles primarily involved in the task (e.g., lower limb muscles during stance). Here, the primary muscle (FCR) was not impacted, rather, the increased contraction occurred in a stabilizer muscle (BB). Notably, anticipatory postural adjustments can be facilitated by stabilizing muscles, and are known to occur before voluntary movements, allowing the body to adjust and maintain stability in response to mechanical forces and environmental changes [54].

Nonetheless, since this study did not measure muscle stiffness, co-contraction, or H-reflexes in the affected muscle (BB), any inferences regarding these factors remain speculative.

Another explanation is that increased demand was placed on the BB to maintain the isometric task as the FCR experienced fatigue. If exercise intensity is submaximal, measurable fatigue can occur without a reduction in task performance, as additional motor units or muscles are recruited to compensate for the ones experiencing fatigue [55]. Here, while additional motor units did not appear to have been recruited by the FCR (as would be indicated by greater EMG), elevations in BB EMG materialized, coinciding with increases in perceived muscle exertion during the VR-C and Post-VR conditions. This suggests a strategy favouring stabilizer muscles including BB to overcome sustained submaximal workloads as the experiment progressed. Notably, BB does not contribute to the primary task of wrist flexion, although participants also maintained a supinated posture, an action to which BB contributes substantially. Furthermore, FCR activity was actively monitored during the study and participants were focused on maintaining it at a consistent level. This may have contributed to their selective elevation of stabilizer exertion while prioritizing FCR stability. Given that the increase in BB activity began alongside the transition into VR and prior to any significant change in perceived exertion, it is likely that participants experienced both a tensing affect related to the sensory manipulation and a compensatory affect related to escalating exertion, which each contributed to the increase in BB EMG while FCR and BR activity remained consistent.

Physiological and psychosocial modulation

Physiological and psychosocial arousal were manipulated by altering visual feedback and the perception of motion within VR. Significant increases in EDA emerged during the VR-C condition suggesting increased sympathetic arousal. Previous studies have demonstrated that threats perceived using VR can induce sympathetic responses including elevations in skin conductance, heart rate, and subjective fear and anxiety perception similar to real environment threatening scenarios [29,31]. Notably, there was no appreciable change in EDA when transitioning between visual environments (real to VR and vice versa). The absence of a significant change upon initial immersion into VR during the VR-BL condition aligns with previous studies, which have reported either minor or no significant changes in sympathetic arousal when entering virtual environments [13,29,56]. Similarly, after exiting the virtual domain, sympathetic activity in the real environment recovered marginally during the Post-VR condition but remained markedly elevated (30.2%) compared to Pre-VR. EDA recovery following a visual manipulation such as VR exposure has not been previously reported on. The invariance of EDA during transitions into and out of VR suggest that altered physiological stress did not factor into any potential neuromuscular adaptations alongside these transitions, instead implicating the influence of the visual manipulation. However, since EDA was significantly elevated alongside the addition of virtual motion during VR-C, sympathetic arousal and/or emotional state may contribute to any accompanying alterations in reflex behaviour *within* VR.

Following a similar pattern, self-reported symptoms of motion sickness were vastly elevated during the VR-C condition compared to all other conditions. This was true when all 16 items on the MSAQ were concatenated as well as when each of the four domains of motion sickness (gastrointestinal, peripheral, central, and sopite-related) were considered

independently. Dissevering this result further reveals three noteworthy findings: i) similar to sympathetic arousal, perceived motion sickness was unaffected by the transition into VR during the VR-BL condition; ii) again in congruence with EDA, motion sickness was strongly elevated when motion was introduced to the VR scene during VR-C; and iii) in contrast to the EDA response, indicators of motion sickness recovered to near baseline levels immediately following return to the real setting during Post-VR. These findings are in partial agreement with previous studies which suggest that VR can induce symptoms of motion sickness (VR sickness). However, in this study, VR in isolation did not evoke VR sickness – symptoms manifested only after movement was perceived in addition to the VR immersion. Furthermore, in contrast to previous work [38], VR sickness evoked in the current study resolved rather quickly following re-entry into the real environment.

Recent studies have discussed the possibility of a significant correlation between motion sickness and EDA [57,58]. This association appears to be appropriate, although the timing of data extraction is an important consideration. Tracking VR sickness using EDA seems most suitable when users are most immersed in the situation rather than at rest or near the starting or end point of the VR exercise [57]. This would explain the coupled responses of EDA and VR sickness we report during VR-C, and their subsequent divergence during recovery in Post-VR.

Neural mechanisms for reflex modulation

Both vibration-induced reflexes (mechanical) and H-reflexes (electrical) are understood to involve short-latency responses that rely on similar spinal circuits, though they differ primarily in their activation mechanisms and location. Vibration-induced reflexes are triggered

by mechanical stretch of the muscle or tendon, engaging the entirety of the Ia afferent pathway via muscle spindle activation. The H-reflex, in contrast, is elicited through direct stimulation of Ia afferent fibers along their journey toward the dorsal horn, therefore bypassing the initial component of the circuit, the muscle spindle. Thus, the H-reflex pathway is incomplete compared to mechanically induced reflexes. Given this distinction, contrasting patterns in responses of these reflexes have been attributed adaptations specific to the muscle spindle such as altered spindle sensitivity, presumably driven by changes in fusimotor activity.

Despite some caution to this approach [10], numerous researchers have proceeded to infer fusimotor activity by evaluating reflexes evoked through mechanical stimulation (e.g., tendon taps or vibration), electrical stimulation (e.g., the H-reflex), or a by comparing both methods within the same subjects and across conditions. In agreement with Burke and colleagues [10], directly comparing mechanical and electrical reflexes *within* a condition to infer fusimotor activity may be considered irresponsible as numerous potential confounding factors likely render neither reflex to be purely monosynaptic; however, independently observing their adaptive patterns *across* conditions enables inferences as to the neural origin of these adaptations to be made with greater confidence. Recent studies suggest that fusimotor drive may play a role in reflex adaptations during conditions of heightened postural threat. For example, Sibley et al. [24] noted reduced H-reflex amplitudes when subjects stood on the edge of a height platform, postulating increased presynaptic inhibition or fusimotor drive. This is supported by subsequent research citing altered spindle sensitivity in conditions of height-induced postural threat via increased stretch [19,27,28], or decreased [13,25,33] or unchanged H-reflexes [19]. A recent study by Hodgson et al. [13] found that H-reflex responses were

reduced in virtual environments simulating standing at varying heights, while vibration-induced reflexes remained stable. They suggested that fusimotor drive may help to maintain spindle sensitivity even when spinal excitability, as measured by the H-reflex, is diminished. Interestingly, in the current study, H-reflex strength was not impacted by transitioning into a stationary VR scene (VR-BL) but was significantly decreased when the virtual rollercoaster was set in motion (VR-C), an adaptation that persisted beyond the transition back into the real environment (Post-VR). Meanwhile, NTV-reflexes remained constant during transition into VR, throughout the VR experience, and after returning from VR. So, the two reflexes manifest very differently during identical conditions prior to and following VR exposure. The contrasting H- and NTV-reflex behaviours arising during VR-C and ensuing through Post-VR supports that neural adaptations specific to muscle spindle circuitry may exist to enable peripheral feedback to compensate for the dampening of overall spinal excitability that seems to arise in conditions of altered visual and/or sympathetic state [13]. This also demonstrates that VR and/or threat induced spinal inhibition may persist for an undetermined period following removal of the stimulus.

Varying hypotheses regarding the role of muscle spindles and the fusimotor system can broadly be divided into two categories: those suggesting a close relationship between fusimotor activity and regular skeletomotor muscle activity ("α-γ co-activation" hypothesis), and those advocating for fusimotor independence [8,9,11,12,59,60]. During α-γ coactivation, coordinated contraction of intrafusal (IF; via γ-MN) and extrafusal (EF; via α-MN) muscle fibers function to maintain spindle sensitivity. This would primarily occur during unloading, or shortening of the spindle-dwelling muscle, though it can also arise during isometric contractions assuming an

increase in EF fibre contraction. In the current study, a change in EMG of the target muscle (FCR) did not accompany the presumed elevation in spindle sensitivity during the VR-C and Post-VR conditions; thus, α-γ co-activation would not explain this result, instead implicating α-γ independence. Importantly, γ-MN activity cannot be directly measured using these methods and can simply be inferred based on indirect measurements using associated neural circuits. However, human microneurography studies have more suitably implied fusimotor independence by recording spindle afferent activity directly. For example, independence has been observed during passive movement tasks requiring focused attention and peripheral feedback [61,62], during small-scale active movements while executing a visuomotor learning task [36,37], and in stationary muscles while preparing for movement [63].

Centrally, dynamic and static γ-MN seem to be independently regulated by specific brain regions including the mesencephalon, basal ganglia, cerebellum, and motor cortex [5]. Peripherally, fusimotor control is shaped by sensory feedback from afferent reflexes originating in muscles, skin, and joints. Descending control enables the nervous system to adapt spindle sensitivity in response to varying motor tasks, sensory inputs, and psychological states, based on volition. Meanwhile, peripheral influences may enable rapid responses to situational factors encountered within the environment. Together, such dynamic modulation likely plays a crucial role in fine-tuning both reflexive and volitional motor actions, ensuring precise motor control during tasks involving postural threat, altered visual feedback, or heightened arousal, independent from EF fibre activity and spinal excitability.

The vestibulospinal tract interacts with other sensory pathways (e.g., visual) to facilitate natural navigation [64] and has been reported to influence H-reflexes in the lower extremity

[65]. This emphasizes the significant role of the vestibular system in influencing spinal excitability by modulating the cortical output to spinal networks during movement. However, its influence is linked to the detection of inertia by the semicircular canals or otolith organs, which are sensitive to body tilt or acceleration. During quiet standing, postural oscillations are not strong enough to trigger reflex modulation by the vestibular system [66]. Thus, seated postures are unlikely to facilitate significant vestibular responses, and the vestibular system likely did not directly influence the findings of the current study.

Limitations and future considerations

A key interpretation of our results is that experiencing visual and emotional perturbations in VR enhances fusimotor activity, which enables the preservation of NTV-reflex strength despite a concurrent increase in spinal inhibition. However, a limitation of our study is that we are making inferences about potential alterations in fusimotor outflow and muscle spindle feedback indirectly by instead measuring reflexive muscle activity. Consequently, we cannot draw definitive conclusions regarding the behavior of muscle spindles or the mechanisms that govern fusimotor control. Future investigations should strengthen these findings by utilizing direct recordings from individual muscle spindles through human microneurography in similar conditions. That said, obtaining direct recordings from single fusimotor neurons in humans is not feasible; therefore, γ-MN activity will still need to be inferred from changes in afferent muscle spindle firing while ensuring that α-MN activity remains stable. This challenge of deducing fusimotor drive from muscle spindle activation is well-recognized [8,9,11,12].

We must also acknowledge that this study cannot make concrete inferences regarding task or location-specific reflex adaptations. Previous studies have explored lower-limb reflex adaptations during stance [13]; here we investigate upper limb reflexes in a seated posture. In the lower limb, muscle reflexes have direct influence on postural control during standing and therefore any miscue can have real and potentially severe consequences. So, there is a genuine threat with direct application to the behaviour of these postural muscles. When encountering a visual or stressful perturbation, especially from a seated position, upper extremity muscles may not necessarily bear the full impact of the threat because compared to lower limb muscles they tend to contribute less to balance or any apparent motor strategy one may employ to circumvent or alleviate the threat or to appropriately navigate their new environment. To fully explore this possibility, it would have been interesting to also assess lower limb reflexes in this study, or for Hodgson and colleagues [13] to have also monitored upper limb reflexes during their study on lower limb reflexes and balance in real and virtual environments. While they reported large changes in lower limb muscle reflexes, which were directly influential on the postural task of quiet standing, it is feasible that upper limb muscle reflexes would have exhibited a smaller adaptation as they were less involved in the task.

Finally, one underappreciated component in this study was the aftereffects of VR immersion. H-reflexes remained suppressed immediately after participants emerged from VR. However, it remains unknown the duration of which these effects persist. So, future studies should explore the recovery of VR aftereffects by reassessing these adaptations at intervals beyond the immediate return to the real environment to determine how long they persist and

whether there is a correlation in the recovery of sympathetic arousal (EDA) and neuromuscular response (H -reflexes).

Conclusion and Future Directions

By elucidating the neural mechanisms that govern motor responses during situations of heightened sympathetic arousal and sensory perturbation such as virtual rollercoaster riding, this research aims to deepen our understanding of human sensorimotor integration and inform the development of interventions aimed at enhancing motor performance and reducing the risk of injury in dynamic environments. Insights gained from this study may have broader implications for fields such as rehabilitation and therapeutics or robotics and prosthetics, where precise control of upper limb movements is essential for optimal performance and safety. It also further highlights the potential for VR as a useful tool for occupational or psychosocial therapies, for example with older adults or in clinical populations such as individuals with anxiety disorders.

The results of this study have important implications for understanding how VR environments affect sensorimotor control and arousal. The sustained suppression of H-reflexes following VR immersion suggest that prolonged VR exposure may have lingering effects on reflex pathways, which could influence motor performance and proprioception in real-world tasks. This has potential applications in both clinical rehabilitation and virtual training environments, where prolonged VR use may impact motor learning or recovery.

Financial Disclosure

This research was funded by Natural Sciences and Engineering Research Council Discovery (RGPIN-2020-06068) and Idea-to-Innovation grants (I2IPJ 548811-2020), as well as a New Frontiers in Research Fund grant (NFRFR-2021-00199) awarded to RMP. DDH was additionally supported through a Natural Sciences and Engineering Research Council Doctoral Postgraduate Scholarship.

Competing Interests

The authors declare no competing interests.

REFERENCES

1. Héroux ME, Butler AA, Robertson LS, Fisher G, Gandevia SC. Proprioception: a new look at an old concept. Journal of Applied Physiology. 2022 Mar 1;132(3):811–4.

2. Peters RM, Dalton BH, Blouin JS, Inglis JT. Precise coding of ankle angle and velocity by human calf muscle spindles. Neuroscience. 2017;349:98-105-98–105.

3. Proske U, Gandevia SC. The proprioceptive senses: Their roles in signaling body shape, body position and movement, and muscle force. Physiological Reviews. 2012;92(4):1651-1697-1651–97.

4. Proske U, Gandevia SC. Kinesthetic senses. Comprehensive Physiology. 2018;8(3):1157-1183-1157–83.

5. Hulliger M. The mammalian muscle spindle and its central control. Reviews of Physiology, Biochemistry and Pharmacology. 1984;101: Volume: 101:1-110-1–110.

6. Macefield VG, Knellwolf TP. Functional properties of human muscle spindles. Journal of neurophysiology. 2018;120(2):452-467-452–67.

7. Matthews PBC. Muscle Spindles and Their Motor Control. Physiological Reviews. 1964 Apr 1;44(2):219–88.

8. Burke D. Crosstalk opposing view: Independent fusimotor control of muscle spindles in humans: there is little to gain. The Journal of Physiology. 2021 May;599(10):2505–8.

9. Burke D. Rebuttal from David Burke. The Journal of Physiology. 2021 May;599(10):2511–2.

10. Burke D, Gandevia SC, McKeon B. The afferent volleys responsible for spinal proprioceptive reflexes in man. The Journal of physiology. 1983;339(1):535-552-535–52.

11. Dimitriou M. Crosstalk proposal: There is much to gain from the independent control of human muscle spindles. Journal of Physiology. 2021;599(10):2501-2504-2501–4.

12. Dimitriou M. Rebuttal from Michael Dimitriou. The Journal of Physiology. 2021 May;599(10):2509–10.

13. Hodgson DD, King JA, Darici O, Dalton BH, Cleworth TW, Cluff T, et al. Visual feedback-dependent modulation of arousal, postural control, and muscle stretch reflexes assessed in real and virtual environments. Front Hum Neurosci [Internet]. 2023 Apr 4 [cited 2024 Nov 8];17. Available from: https://www.frontiersin.org/journals/human-neuroscience/articles/10.3389/fnhum.2023.1128548/full

14. Mildren RL, Peters RM, Hill AJ, Blouin JS, Carpenter MG, Inglis JT. Frequency characteristics of human muscle and cortical responses evoked by noisy Achilles tendon vibration. Journal of Applied Physiology. 2017 May 1;122(5):1134–44.

15. Mildren RL, Peters RM, Carpenter MG, Blouin JS, Inglis JT. Soleus single motor units show stronger coherence with Achilles tendon vibration across a broad bandwidth relative to medial gastrocnemius units while standing. Journal of Neurophysiology. 2019 Nov 1;122(5):2119–29.

16. Pierrot-Deseilligny E, Burke DJ. The Circuitry of the Human Spinal Cord : Its Role in Motor Control and Movement Disorders. Cambridge University Press; 2005.

17. Theodosiadou A, Henry M, Duchateau J, Baudry S. Revisiting the use of Hoffmann reflex in motor control research on humans. European Journal of Applied Physiology. 2023;123(4):695-710-695–710.

18. Zehr EP. Considerations for use of the Hoffmann reflex in exercise studies. Eur J Appl Physiol. 2002 Apr;86(6):455–68.

19. Horslen BC, Murnaghan CD, Inglis JT, Chua R, Carpenter MG. Effects of postural threat on spinal stretch reflexes: evidence for increased muscle spindle sensitivity? Journal of Neurophysiology. 2013 Aug 15;110(4):899–906.

20. Nafati G, Rossi-Durand C, Schmied A. Proprioceptive control of human wrist extensor motor units during an attention-demanding task. Brain Research. 2004 Aug;1018(2):208–20.

21. Cattagni T, Martin A, Scaglioni G. Is spinal excitability of the triceps surae mainly affected by muscle activity or body position? Journal of Neurophysiology. 2014 Jun 15;111(12):2525–32.

22. Cecen S, Niazi IK, Nedergaard RW, Cade A, Allen K, Holt K, et al. Posture modulates the sensitivity of the H-reflex. Experimental Brain Research. 2018;236:829-835-829–35.

23. Chalmers GR. Modulation of tendon tap reflex activation of soleus motor neurons with reduced stability tandem stance. Human movement science. 2019;64:274-282-274–82.

24. Sibley KM, Carpenter MG, Perry JC, Frank JS. Effects of postural anxiety on the soleus H-reflex. Human movement science. 2007;26(1):103-112-103–12.

25. Llewellyn M, Yang JF, Prochazka A. Human H-reflexes are smaller in difficult beam walking than in normal treadmill walking. Exp Brain Res [Internet]. 1990 Dec [cited 2025 Jan 12];83(1). Available from: http://link.springer.com/10.1007/BF00232189

26. McIlroy WE, Bishop DC, Staines WR, Nelson AJ, Maki BE, Brooke JD. Modulation of afferent inflow during the control of balancing tasks using the lower limbs. Brain Research. 2003 Jan;961(1):73–80.

27. Davis JR, Horslen BC, Nishikawa K, Fukushima K, Chua R, Inglis JT, et al. Human proprioceptive adaptations during states of height-induced fear and anxiety. Journal of Neurophysiology. 2011;106(6):3082-3090-3082–90.

28. Horslen BC, Zaback M, Inglis JT, Blouin J, Carpenter MG. Increased human stretch reflex dynamic sensitivity with height-induced postural threat. The Journal of Physiology. 2018 Nov;596(21):5251–65.

29. Cleworth TW, Horslen BC, Carpenter MG. Influence of real and virtual heights on standing balance. Gait & Posture. 2012 Jun;36(2):172–6.

30. Cleworth TW, Chua R, Inglis JT, Carpenter MG. Influence of virtual height exposure on postural reactions to support surface translations. Gait & Posture. 2016 Jun;47:96–102.

31. Meehan M, Insko B, Whitton M, Brooks FP. Physiological measures of presence in stressful virtual environments. ACM Trans Graph. 2002 Jul;21(3):645–52.

32. Nielsen EI, Cleworth TW, Carpenter MG. Exploring emotional-modulation of visually evoked postural responses through virtual reality. Neuroscience Letters. 2022 Apr;777:136586.

33. Grosprêtre S, Eon P, Marcel-Millet P. Virtual reality does not fool the brain only: spinal excitability changes during virtually simulated falling. Journal of Neurophysiology. 2023;129(2):368-379-368–79.

34. Hoffman MA, Koceja DM. The effects of vision and task complexity on Hoffmann reflex gain. Brain research. 1995;700(1–2):303-307-303–7.

35. Hoshiyama M, Koike Y, Watanabe S. Effect of optokinetic stimulation on the H reflex in humans. The Japanese Journal of Physiology. 1994;44(1):67-73-67–73.

36. Dimitriou M. Enhanced muscle afferent signals during motor learning in humans. Current Biology. 2016;26(8):1062-1068-1062–8.

37. Jones KE, Wessberg J, Vallbo Å. Proprioceptive feedback is reduced during adaptation to a visuomotor transformation: preliminary findings. Neuroreport. 2001;12(18):4029-4033-4029–33.

38. Stanney KM, Kennedy RS, Drexler JM, Harm DL. Motion sickness and proprioceptive aftereffects following virtual environment exposure. Applied Ergonomics. 1999 Feb 1;30(1):27–38.

39. Szpak A, Michalski SC, Saredakis D, Chen CS, Loetscher T. Beyond Feeling Sick: The Visual and Cognitive Aftereffects of Virtual Reality. IEEE Access. 2019;7:130883–92.

40. Mercan M, Kuruoğlu R. The H-reflex study of the flexor carpi radialis muscle in healthy individuals. Front Neurol. 2024 Nov 19;15:1462882.

41. Palmieri RM, Ingersoll CD, Hoffman MA. The hoffmann reflex: methodologic considerations and applications for use in sports medicine and athletic training research. J Athl Train. 2004 Jul;39(3):268–77.

42. Gianaros PJ, Muth ER, Mordkoff JT, Levine ME, Stern RM. A questionnaire for the assessment of the multiple dimensions of motion sickness. Aviat Space Environ Med. 2001 Feb;72(2):115–9.

43. Borg G. An introduction to Borg's RPE-scale. Mouvement Publications. 1985;

44. Sabbahi MA, Khalil M. Segmental H-reflex studies in upper and lower limbs of healthy subjects. Arch Phys Med Rehabil. 1990 Mar;71(3):216–22.

45. Society for Psychophysiological Research Ad Hoc Committee on Electrodermal Measures. Publication recommendations for electrodermal measurements. Psychophysiology. 2012 Aug;49(8):1017–34.

46. Noble BJ, Borg GA, Jacobs I, Ceci R, Kaiser P. A category-ratio perceived exertion scale: relationship to blood and muscle lactates and heart rate. Med Sci Sports Exerc. 1983;15(6):523–8.

47. Field AP. Discovering statistics using IBM SPSS statistics. Fourth edition. South Asian adaptation. New Delhi, India: SAGE Publications; 2020.

48. Cohen J. Statistical Power Analysis for the Behavioral Sciences [Internet]. 0 ed. Routledge; 2013 [cited 2025 Jan 12]. Available from: https://www.taylorfrancis.com/books/9781134742707

49. Morasso PG, Sanguineti V. Ankle Muscle Stiffness Alone Cannot Stabilize Balance During Quiet Standing. Journal of Neurophysiology. 2002 Oct 1;88(4):2157–62.

50. Carpenter M, Frank J, Silcher C, Peysar G. The influence of postural threat on the control of upright stance. Experimental Brain Research. 2001 May 16;138(2):210–8.

51. Carpenter MG, Frank JS, Silcher CP. Surface height effects on postural control: A hypothesis for a stiffness strategy for stance*. VES. 1999 Aug 1;9(4):277–86.

52. Azevedo TM, Volchan E, Imbiriba LA, Rodrigues EC, Oliveira JM, Oliveira LF, et al. A freezing-like posture to pictures of mutilation. Psychophysiology. 2005 May;42(3):255–60.

53. Facchinetti LD, Imbiriba LA, Azevedo TM, Vargas CD, Volchan E. Postural modulation induced by pictures depicting prosocial or dangerous contexts. Neuroscience Letters. 2006 Dec;410(1):52–6.

54. Cavallari P, Bolzoni F, Bruttini C, Esposti R. The Organization and Control of Intra-Limb Anticipatory Postural Adjustments and Their Role in Movement Performance. Front Hum Neurosci [Internet]. 2016 Oct 19 [cited 2025 Feb 6];10. Available from: http://journal.frontiersin.org/article/10.3389/fnhum.2016.00525/full

55. Taylor JL, Gandevia SC. A comparison of central aspects of fatigue in submaximal and maximal voluntary contractions. Journal of Applied Physiology. 2008 Feb;104(2):542–50.

56. Simeonov PI, Hsiao H, DotsonM BW, Ammons DE. Height Effects in Real and Virtual Environments. Hum Factors. 2005 Jun;47(2):430–8.

57. Seong S, Park J. Tracking motion sickness in dynamic VR environments with EDA signals. International Journal of Industrial Ergonomics. 2024 Jan;99:103543.

58. Smyth J, Birrell S, Woodman R, Jennings P. Exploring the utility of EDA and skin temperature as individual physiological correlates of motion sickness. Applied Ergonomics. 2021 Apr;92:103315.

59. Prochazka A. Sensory control of normal movement and of movement aided by neural prostheses. Journal of Anatomy. 2015 Aug;227(2):167–77.

60. Prochazka A, Ellaway P. Sensory Systems in the Control of Movement. In: Prakash YS, editor. Comprehensive Physiology [Internet]. 1st ed. Wiley; 2012 [cited 2025 Feb 6]. p. 2615–27. Available from: https://onlinelibrary.wiley.com/doi/10.1002/cphy.c100086

61. Hospod V, Aimonetti JM, Roll JP, Ribot-Ciscar E. Changes in human muscle spindle sensitivity during a proprioceptive attention task. Journal of Neuroscience. 2007;27(19):5172-5178-5172–8.

62. Ribot-Ciscar E, Hospod V, Roll JP, Aimonetti JM. Fusimotor drive may adjust muscle spindle feedback to task requirements in humans. Journal of neurophysiology. 2009;101(2):633-640-633–40.

63. Papaioannou S, Dimitriou M. Goal-dependent tuning of muscle spindle receptors during movement preparation. Science Advances. 2021;7(9):0401–0401.

64. Cheng Z, Gu Y. Vestibular System and Self-Motion. Front Cell Neurosci. 2018 Nov 22;12:456.

65. Nepveu JF, Mikhail Y, Pion CH, Gossard JP, Barthélemy D. Assessment of vestibulocortical interactions during standing in healthy subjects. Schwenkreis P, editor. PLoS ONE. 2020 Jun 4;15(6):e0233843.

66. Nashner LM, Shupert CL, Horak FB, Black FO. Chapter 33 Organization of posture controls: an analysis of sensory and mechanical constraints. In: Progress in Brain Research [Internet]. Elsevier; 1989 [cited 2025 Feb 6]. p. 411–8. Available from: https://linkinghub.elsevier.com/retrieve/pii/S0079612308622372

Figure 1

Click here to access/download;Figure;Figure 1.tif

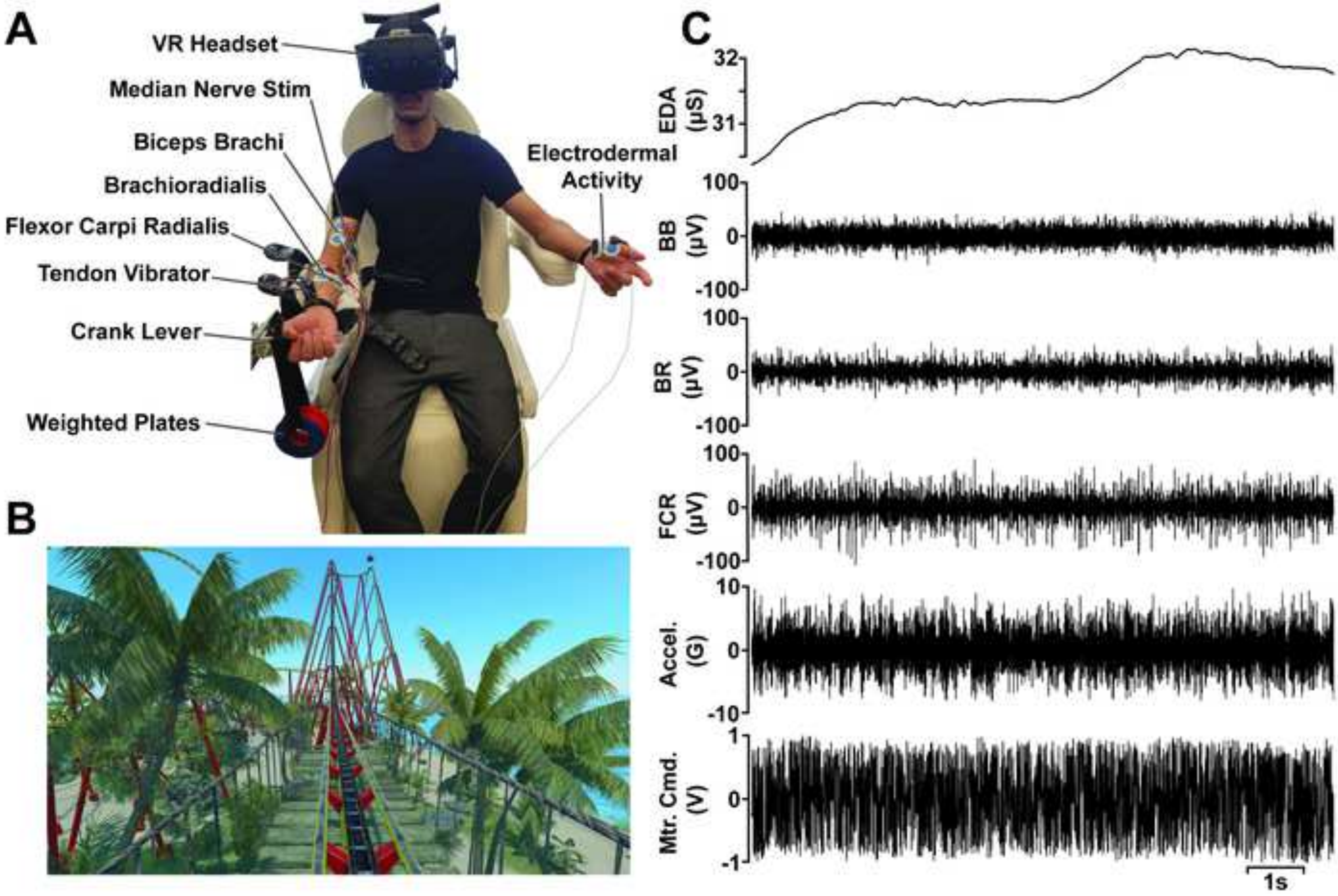

Figure 2

Click here to access/download;Figure;Figure 2.tif

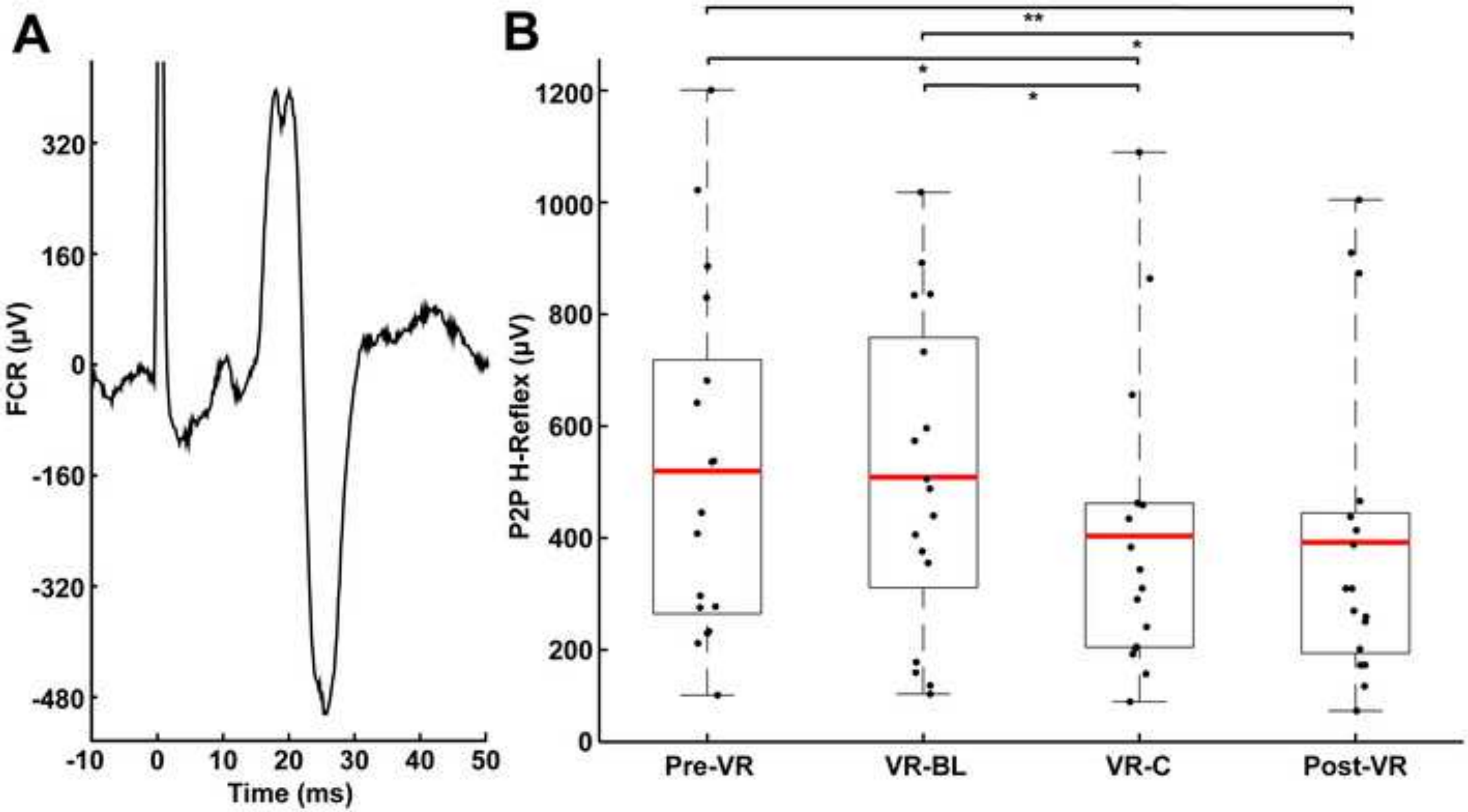

Figure 3

Click here to access/download;Figure;Figure 3.tif

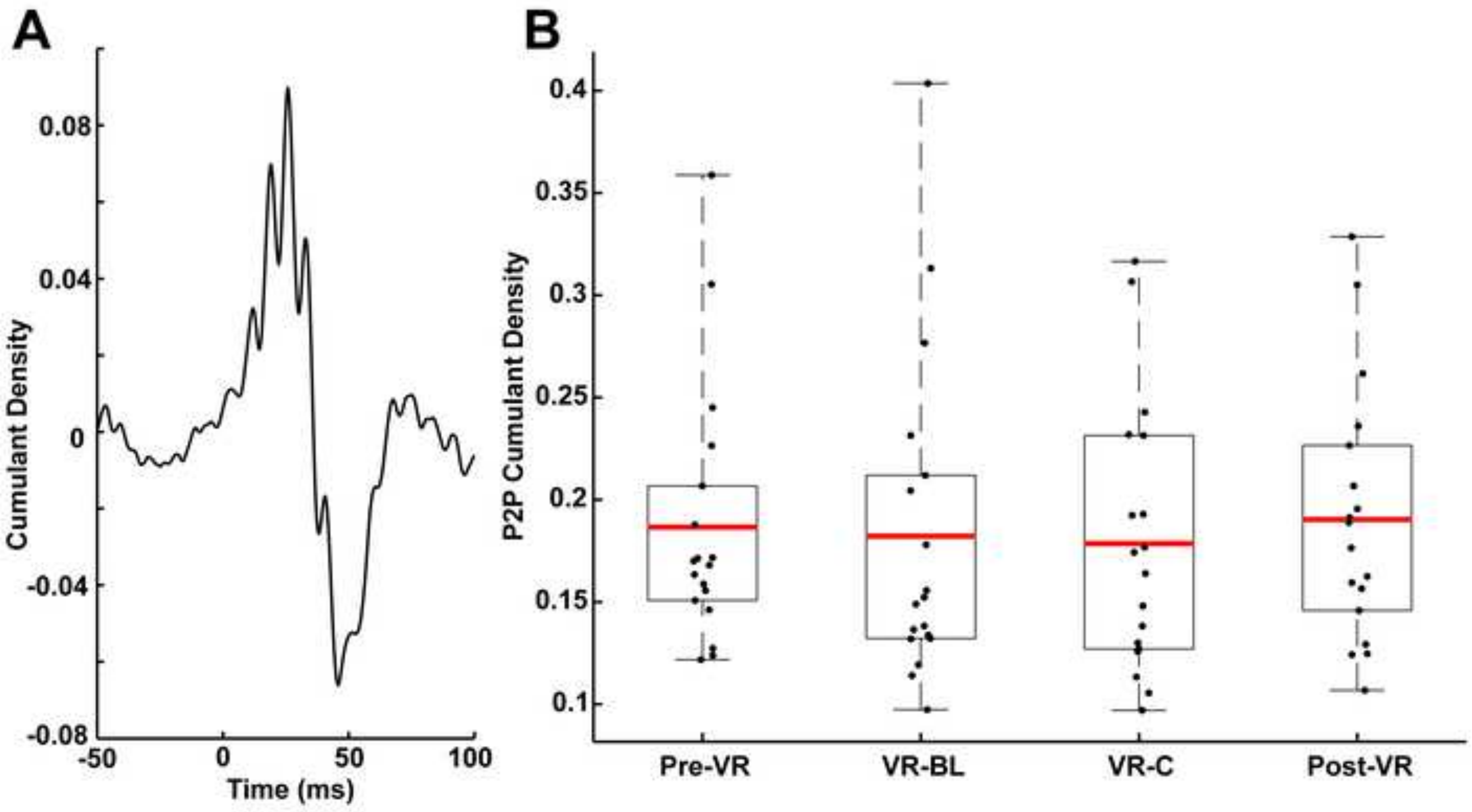

Click here to access/download;Figure;Figure 4.tif

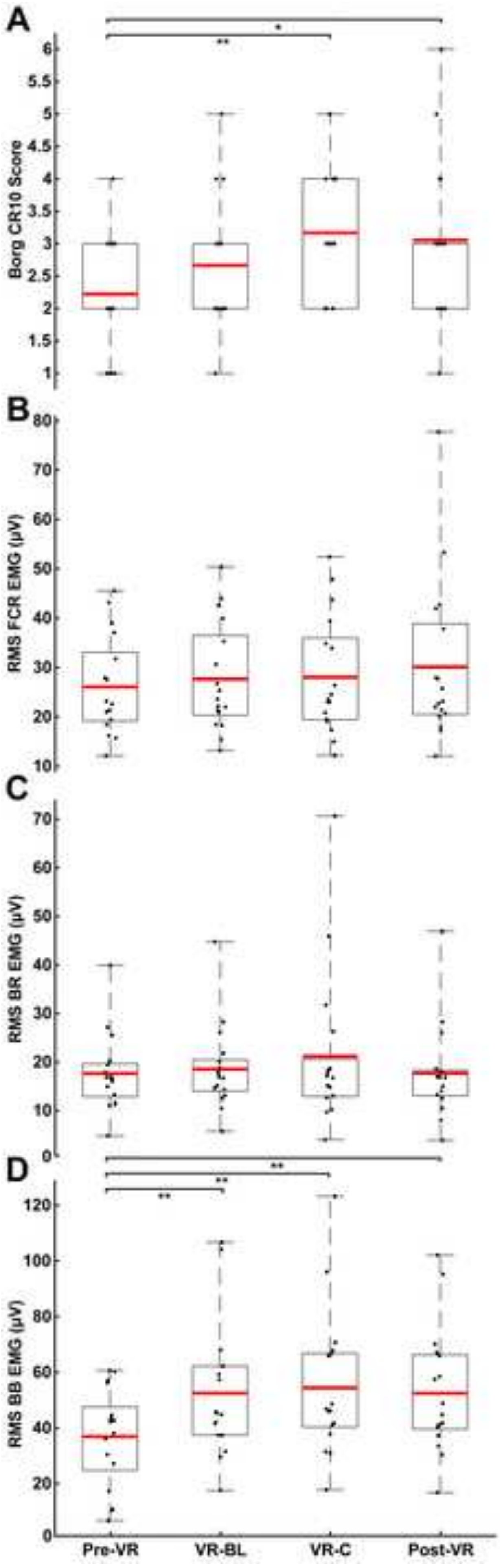

Figure 5

Click here to access/download;Figure;Figure 5.tif

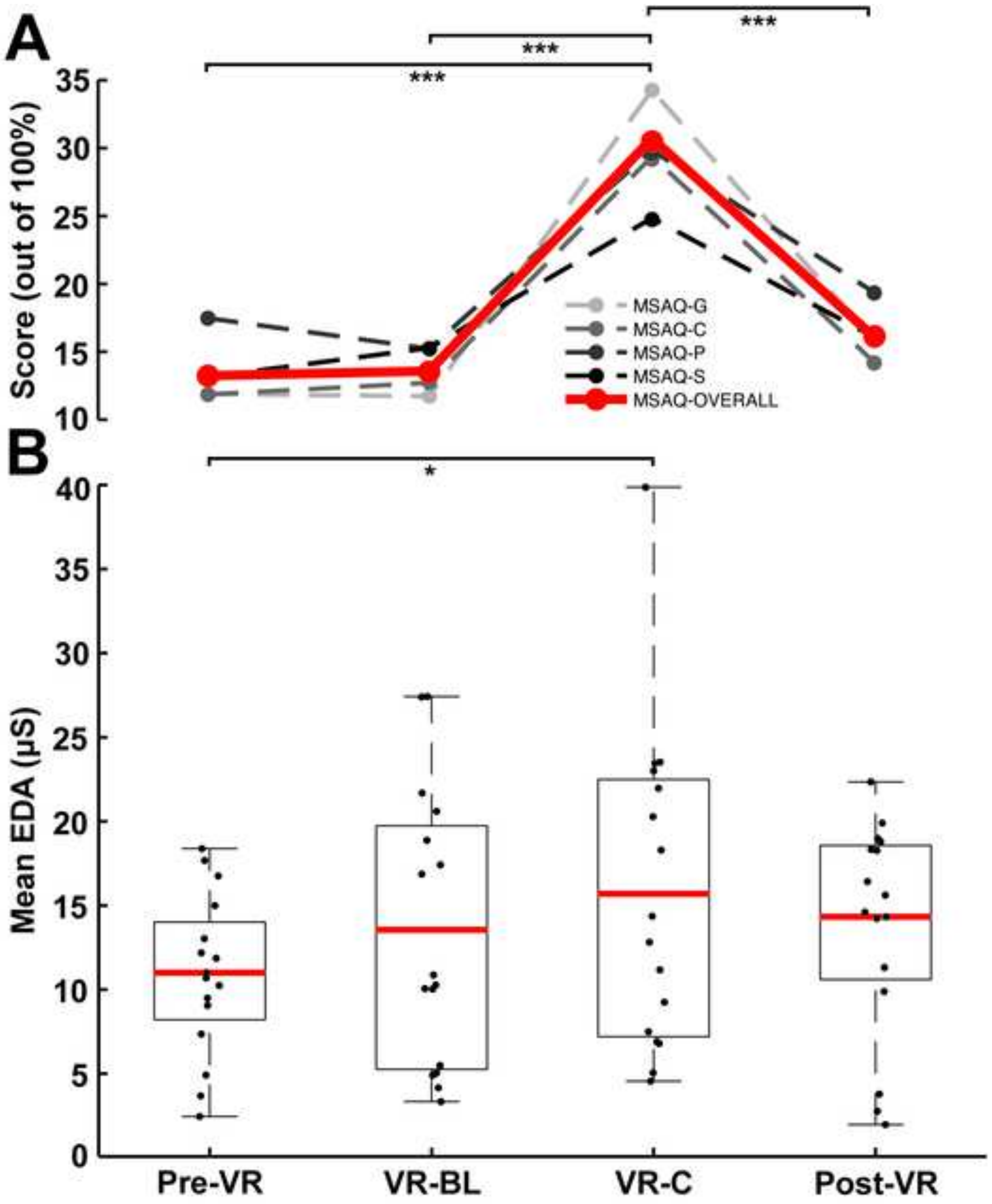

Supplement: Attachment — Submitted filename: PONE-D-25-07247_Comments.pdf [file pone.0330129.s002.pdf]
